# Detection of *Borrelia* *burgdorferi* Sensu Lato and Relapsing Fever *Borrelia* in Feeding *Ixodes* Ticks and Rodents in Sarawak, Malaysia: New Geographical Records of *Borrelia yangtzensis* and *Borrelia miyamotoi*

**DOI:** 10.3390/pathogens9100846

**Published:** 2020-10-15

**Authors:** Alice C. C. Lau, Yongjin Qiu, Mohamed Abdallah Mohamed Moustafa, Ryo Nakao, Michito Shimozuru, Manabu Onuma, Jayasilan Mohd-Azlan, Toshio Tsubota

**Affiliations:** 1 Laboratory of Wildlife Biology and Medicine, Department of Environmental Veterinary Sciences, Faculty of Veterinary Medicine, Hokkaido University, Sapporo 060-0818, Japan; alicelau.cc@vetmed.hokudai.ac.jp (A.C.C.L.); shimozuru@vetmed.hokudai.ac.jp (M.S.); 2 Hokudai Center for Zoonosis Control in Zambia, Research Center for Zoonosis Control, Hokkaido University, Sapporo 001-0020, Japan; 3 Laboratory of Parasitology, Department of Disease Control, Faculty of Veterinary Medicine, Hokkaido University, Sapporo 060-0818, Japan; m.abdallah@vetmed.hokudai.ac.jp (M.A.M.M.); ryo.nakao@vetmed.hokudai.ac.jp (R.N.); 4 Department of Animal Medicine, Faculty of Veterinary Medicine, South Valley University, Qena 83523, Egypt; 5 Ecological Risk Assessment and Control Section, Center for Environmental Biology and Ecosystem Studies, National Institute for Environmental Studies, Tsukuba 305-806, Japan; monuma@nies.go.jp; 6 Institute of Biodiversity and Environmental Conservation, University Malaysia Sarawak, Kota Samarahan, Sarawak 94300, Malaysia; azlan@unimas.my

**Keywords:** *Borrelia miyamotoi*, *Borrelia yangtzensis*, *Ixodes granulatus*, rodent, Sarawak, Malaysia

## Abstract

Members of the *Borrelia burgdorferi* sensu lato (Bbsl) complex are etiological agents of Lyme disease (LD), and *Borrelia miyamotoi* is one of the relapsing fever *Borrelia* (RFB). Despite the serological evidence of LD in Malaysia, there has been no report from Sarawak, Malaysian Borneo. Thus, this study aimed to detect and characterize *Borrelia* in rodents and *Ixodes* ticks from primary forests and an oil palm (OP) plantation in Sarawak. *Borrelia yangtzensis* (a member of the Bbsl complex) was detected in 43.8% (14/32) of *Ixodes granulatus*; most of the positive ticks were from the OP plantation (13/14). Out of 56 rodents, *B. yangtzensis* was detected in four *Rattus* spp. from the OP plantation and *B. miyamotoi* was detected in one rodent, *Sundamys muelleri*, from the primary forest. Further, the positive samples of *B. yangtzensis* were randomly selected for multilocus sequence analysis (MLSA). The MLSA results of successfully amplified tick samples revealed a clustering with the sequences isolated from Japan and China. This study is the first evidence of *B. miyamotoi*, a known human pathogen in Malaysia, and *B. yangtzensis*, which is circulating in ticks and rodents in Sarawak, Malaysian Borneo, and presenting a new geographical record of the *Borrelia* spp.

## 1. Introduction

Members of the *Borrelia burgdorferi* sensu lato (Bbsl) complex are etiological agents of Lyme disease (LD), and *Borrelia miyamotoi* is one of the relapsing fever *Borrelia* (RFB) [1]. There are currently more than 20 genospecies in the Bbsl complex reported worldwide [2]. The natural transmission cycles of Bbsl are maintained between their vertebrate reservoir hosts and ixodid (hard) ticks (Acari: Ixodidae) as the vector [3,4]. High incidences of LD and high occurrences of its causative bacterium have been continuously reported since the discovery of LD spirochetes in 1982 in North America and later in 1983 in Europe [4]. In North America, several Bbsl genospecies, including *B. burgdorferi* sensu stricto (Bbss), *B. afzelii*, *B. garinii*, *B. californiensis*, *B. bissettiae*, *B. kurtenbachii*, *B. mayonii*, and *B. spielmanii* are known to be responsible for LD [5,6,7,8,9]. While in Europe, Bbss, *B. garinii*, *B. afzelii*, and *B. bavariensis* are the predominant causative bacterium for LD cases [10]. LD remains one of the most important infectious diseases in these two continents. Moreover, this disease is also endemic in east Asian countries as there have been reports from China, Japan, Taiwan, South Korea, and Russia [11,12].

Relapsing fever *Borrelia* (RFB) is categorized into three genetic groups: New World RFB, Old World RFB, and RFB harbored by ixodid ticks. Among them, New World and Old World RFB are transmitted by soft ticks and louse [13] and are endemic to the African countries, Middle East, Central Asia, southern Europe, and North America [13,14]. On the other hand, RFB harbored by ixodid ticks, including *B. miyamotoi*, *B. lonestari*, and *B. theileri*, are mainly transmitted by the genus *Ixodes*, *Amblyomma*, and *Rhipicephalus* ticks, respectively [15,16,17]. Among these three, *B. miyamotoi* is pathogenic to humans [18], and *B. theileri* is responsible for bovine borreliosis [19]. *B. miyamotoi* infection in humans was first reported in Russia in 2011, and, since then, cases have been documented in the United States, Europe, Japan, and northeastern China [18,20,21,22,23].

Malaysia is geographically located in the Southeast Asian region and is divided into Peninsular Malaysia and Malaysian Borneo. Malaysian Borneo consists of Sarawak and Sabah states and is located in Borneo Island with two other countries: Brunei and Indonesia. Sarawak is the largest state in Malaysia and has tropical geography and equatorial climate. These climatic conditions of relatively high daily average temperature and all-year-round humidity could be ideal for tick survival [24]. Further, Sarawak has undergone massive forest degradation and fragmentation, mainly because of logging activities and oil palm plantations [25,26], and land conversion has been significantly related to increasing emerging or re-emerging zoonotic diseases, including vector-borne diseases [27,28].

To date, the number of studies on tick-borne pathogens, such as *Borrelia*, has been limited and largely overlooked in Malaysia. Only a few studies on *Borrelia* spp. were conducted in Peninsular Malaysia, where *Borrelia* spp., belonging to the RFB group, were detected in the *Haemaphysalis hystricis* ticks collected from wild boar carcasses [29]. Additionally, *Borrelia* spp., closely related to *B. yangtzensis* (a member of Bbsl), were reported from *Ixodes granulatus* collected from rodents [30]. Moreover, antibodies against LD were detected from both the urban and indigenous people communities; this provided the serological evidence of borrelial infection in humans [31,32]. Nevertheless, no study on *Borrelia* has been conducted in Malaysian Borneo or Sarawak, so its status is unknown.

Most of the prior studies conducted in Peninsular Malaysia focused on tick surveillance, and little is known about the occurrence of these etiological agents in most parts of Malaysia, including Sarawak. Hence, in this study, we investigated the presence of *Borrelia* in rodents and *Ixodes* ticks collected in Sarawak, Malaysian Borneo.

## 2. Results

### 2.1. Identification of Rodent and Tick Species

**Figure 1:**
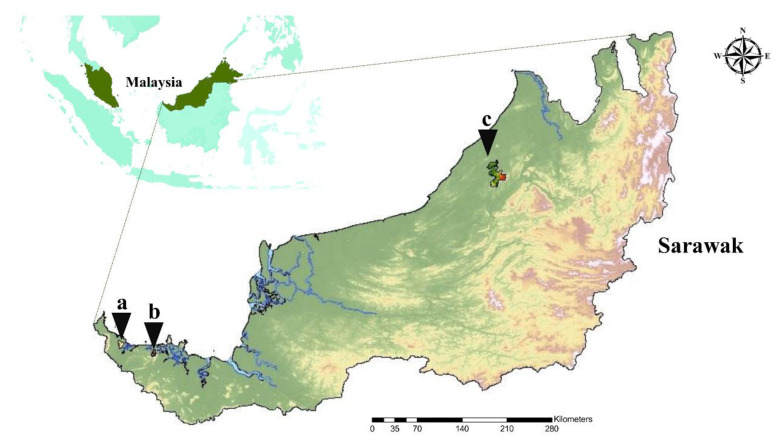
Map of the sampling sites. (**a**) Gunung Gading National Park, (**b**) Kubah National Park, and (**c**) oil palm plantation.

Overall, we trapped 97 rodents from two primary forests, Gunung Gading National Park (GGNP) and Kubah National Park (KNP), and an oil palm (OP) plantation, out of which 56 rodents were selected and used for this study based on the research permissions (Figure 1). Sample identity was sampling site abbreviation (KNP-, GGNP-, and OP-), followed by a numerical assignment. The morphological and molecular identification of the rodents revealed 4 *Leopodamys sabanus*, 2 *Maxomys rajah*, 2 *M. whiteheadi*, 45 *Rattus* spp., and 3 *Sundamys muelleri* (Table 1). The rodents that were morphologically assigned to the *Rattus* spp., *R. tiomanicus*, and *R. tanezumi*, were collectively grouped as one *Rattus* spp., as they were not molecularly identified as a single species.

A total of 32 *Ixodes* ticks (22 females, 5 nymphs, and 5 larvae) were collected from the rodents and molecularly identified as *I. granulatus* (Table 1). All the *I. granulatus* ticks were engorged or semi-engorged, except for one larva from the OP plantation. A similar system was also applied for tick sample identity, by using IG as the acronym for *I. granulatus*.

### 2.2. Detection of the Borrelia spp.

Out of the 56 rodent samples, four *Rattus* spp. (Sample IDs: OP-007, -014, -018, and -033) from the OP plantation and one *S. muelleri* (Sample ID: GGNP-04) from the GGNP were positive for the borrelial flagellin gene (*flaB*) in a polymerase chain reaction (PCR) (Table 1). The prevalence of *Borrelia* spp. in rodents from GGNP, KNP, and the OP plantation were 16.7% (1/6), 0% (0/7), and 8.9% (4/45), respectively (Table 1). Subsequent analysis revealed that sequences from these five samples were different from each other. The sequences from OP-007 and OP-014 showed 100% (300/300 bp) and 95% (285/300 bp) identity, respectively, with *B. yangtzensis* in *I. granulatus* extracted from rodent *Niviventer fulvescens* in China (EU135602). The sequence from OP-018 showed 100% (300/300 bp) identity with *B. valasiana*-related genospecies from rodent *Suncus murinus* in Japan (AB091710). The sequence from OP-033 showed 99.3% (298/300 bp) identity with *B. valasiana*-related genospecies from rodent *Apodemus agrarius* in China (AB022136). Finally, the sequence from GGNP-04 showed 100% (294/294 bp) identity with *B. miyamotoi* from *I. nipponensis* in South Korea (MH102393). For further characterization of the *Borrelia* spp., subsequent nested PCRs targeting the borrelial 16S ribosomal DNA (rDNA) were carried out and successful in four of the samples (OP-014, -018, -033, and GGNP-04). The sequence from OP-014 showed 99.6% (1348/1354 bp) identity with *B. yangtzensis* from *H. longicornis* in China (EU135595). The sequence from OP-018 showed 99.7% (1350/1354 bp) identity with *B. valasiana*-related genospecies from rodent *A. agrarius* in China (AB022141). The sequence from OP-033 showed 99.9% identity with *B. valasiana*-related genospecies from South Korea (U44938). Finally, the sequence from GGNP-04 showed 99.8% (1352/1355 bp) identity with *B. miyamotoi* from a febrile patient in Russia (CP037471).

Out of 32 *I. granulatus* samples, 14 samples (43.8%) were positive for *flaB*-PCR. The 14 samples included 1 female from KNP and 10 females, 1 nymph, and 2 larvae from the OP plantation (Table 1). None of the *I. granulatus* samples from GGNP were positive for *flaB*-PCR. In addition, only one positive *I. granulatus* sample (Sample ID: IG-218) was harvested from the Bbsl positive rodent, OP-033. Among the three sampling sites, the OP plantation recorded the highest number of tested samples and the highest prevalence of positive samples (72.2%; 13/18; Table 1). The details for the sequencing results of both *flaB* and 16S rDNA were shown in Table 2. From the sequence analysis, six *I. granulatus* (Sample IDs: IG-204, -206, -208, -213, -217, and -218) had an identical sequence of *flaB*, i.e., 100% (300/300 bp) identity with *B. yangtzensis* (EU135602). This sequence was also identical to that from the rodent sample, OP-007. Additionally, sequences from sample IDs: IG-214, -216, -220, -221, -222, and -228) were identical with Bbsl sequences from *I. granulatus* in Taiwan (HM853004), China (MG717513 and MG717514), and Malaysia (LT969779) (Table 2). Finally, the sequences from sample IDs: IG-215 and -219 showed 99.3% (298/300 bp) and 100% (300/300 bp) identity, respectively, with *B. valaisiana*-related genospecies (AB091710), and the sequence from IG-215 was identical to that from the rodent sample, OP-018. Additionally, 7 out of the 14 *I. granulatus* samples with a positive *flaB*-PCR were successfully sequenced for 16S rDNA. The sequences showed high similarity with *B. yangtzensis* from China (EU135595 and EU135598) and South Korea (L39080) and with *B. valaisiana*-related genospecies from China (AB022140 and AB022141) (Table 2). Similarly, the same sequences were also detected in both tick and rodent samples. The sequence from IG-219 had an identical sequence to the rodent sample, OP-018.

### 2.3. Phylogenetic Analysis

**Figure 2:**
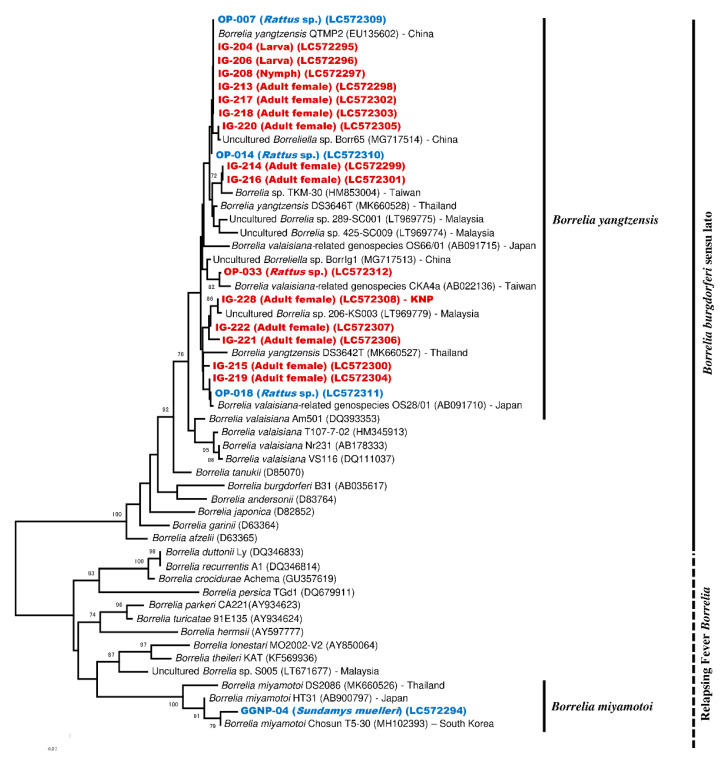
Phylogenetic tree based on *flaB* sequences of the *Borrelia* species. The phylogenetic tree was constructed in MEGA version X by the Neighbor-Joining model with Kimura-2 parameter and 1000 bootstrap replications. The sequences from ticks and rodents obtained in this study are shown in red and blue, respectively. All positive tick samples were collected from an oil palm (OP) plantation; the location is not indicated except for one sample from Kubah National Park (KNP). The individual rodent IDs are shown with the sampling sites: Gunung Gading National Park (GGNP) or the OP plantation.

**Figure 3:**
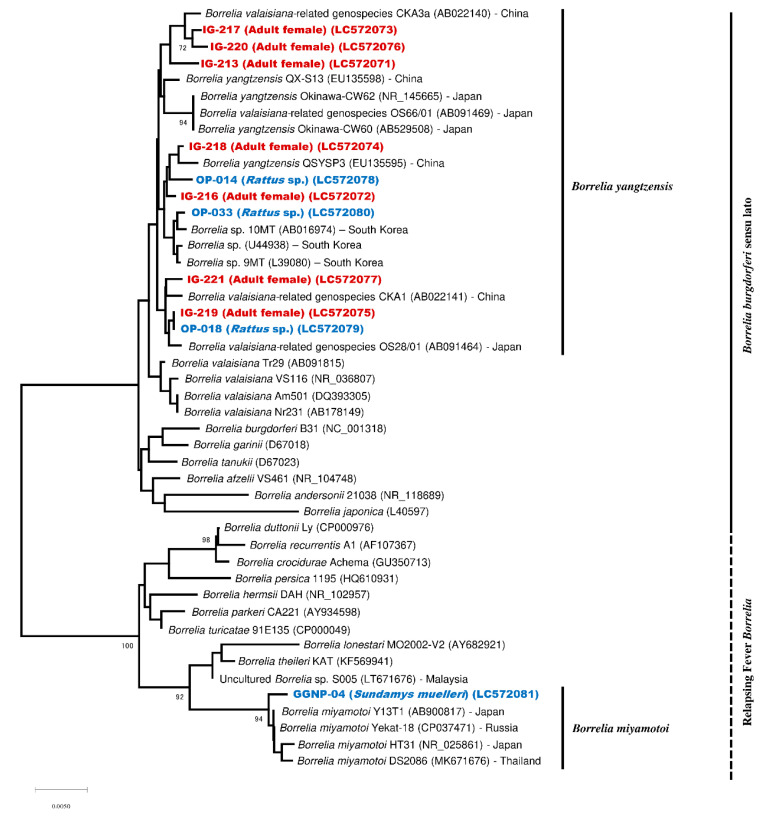
Phylogenetic tree based on 16S rDNA sequences of the *Borrelia* species. The phylogenetic was constructed in MEGA version X by the Neighbor-Joining model with Kimura-2parameter and 1000 bootstrap replications. The sequences from ticks and rodents obtained in the present study are shown in red and blue, respectively. All samples were collected from an oil palm (OP) plantation, except one sample, which was from Gunung Gading National Park (GGNP).

Collectively, 19 samples (5 rodents and 14 *I. granulatus*) were positive for *flaB*-PCR and were included in the phylogenetic tree construction. Out of 19 samples, 18 were assigned to the clade of Bbsl and clustered together with *B. yangtzensis* or *B. valaisiana*-related genospecies (Figure 2). The remaining rodent sample (GGNP-04) was assigned to the RFB and clustered together with *B. miyamotoi* (Figure 2). In addition, the phylogenetic tree based on the 16S rDNA sequences revealed the consistent clustering, as observed in *flaB* (Figure 3).

### 2.4. Multilocus Sequence Analysis of the Borrelia spp.

**Figure 4:**
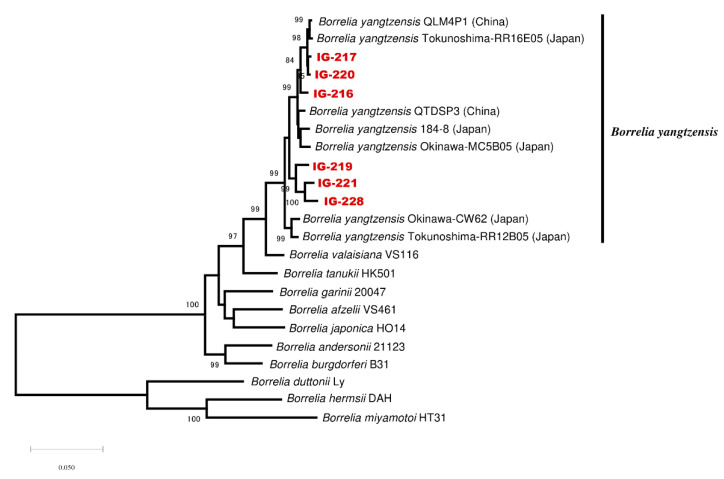
Phylogenetic inference of the *Borrelia burgdorferi* sensu lato. The samples are shown in red. The sequences obtained from eight housekeeping genes were trimmed and concatenated in the order of *clpA*, *clpX*, *nifS*, *pepX*, *pyrG*, *recG*, *rplB*, and *uvrA*, according to the *Borrelia* PubMLST database. The maximum likelihood model was used with 1000 bootstrap replications for the phylogenetic construction in MEGA version X.

All *I. granulatus* samples included for the multilocus sequence analysis (MLSA) were successfully amplified for the eight housekeeping genes. In the phylogenetic tree based on the concatenated MLSA genes, the *Borrelia* spp. from *I. granulatus* were located in the clade of *B. yangtzensis* (Figure 4). This trend was also confirmed in the other phylogenetic trees based on *flaB* and 16S rDNA (Figure 2 and Figure 3). For the four rodent samples included in MLSA, a minimum of two (1/4), four (2/4), and six (1/4) housekeeping genes were successfully amplified. Therefore, the phylogenetic inferences of the *Borrelia* species in both rodent and tick samples were done on a per-gene basis. The phylogenetic analysis based upon each housekeeping gene showed that the rodent samples (OP-007, -014, -018, and -033) were located in the clade of *B. yangtzensis* with *I. granulatus* from this study (Figure S1).

## 3. Discussion

We investigated *Borrelia* spp. in *I. granulatus* and rodents, *L. sabanus*, *M. rajah*, *M. whiteheadi*, *Rattus* spp., and *S. muelleri* from primary forests (GGNP and KNP) and an OP plantation in Sarawak, Malaysia. We identified *B. yangtzensis* from *I. granulatus* and *Rattus* spp. and *B. miyamotoi* from *S. muelleri*. This study is the first evidence of *B. miyamotoi* in Malaysia and *B. yangtzensis* in Sarawak, Malaysian Borneo.

*Borrelia yangtzensis* detected from *I. granulatus* in this study was formerly known as *B. valaisiana*-related genospecies since phylogenetic inferences showed a close relation but a clear distinction to *B. valaisiana* (a member of the Bbsl complex in Europe) [33]. However, unlike *B. valaisiana* that utilize birds as the reservoir host and *Ixodes* ticks as the vector [33,34], *B. yangtzensis* is maintained and transmitted through a natural infection cycle between rodents and *Ixodes* ticks [35]. Isolations of *B. yangtzensis* from different rodent species were recorded from *Rattus* spp., *S. murinus*, *Mus* spp., and *A. agrarius* in Japan, China, and Taiwan [35,36,37], as well as from different *Ixodes* tick species, such as *I. nipponensis* in South Korea and *I. granulatus* in Japan and China [35,38,39]. Recently, a *Borrelia* sp. closely related to *B. yangtzensis* was detected in Peninsular Malaysia from *I. granulatus* collected from different rodent species [30]. Takhampunya et al. [40] also identified *B. yangtzensis* from one rodent and two tick pools of *Ixodes* spp. collected from rodents in northern Thailand. This is similar to our study, as we detected *B. yangtzensis* from *Rattus* spp. and *I. granulatus* for the first time in Sarawak. These results suggest that *B. yangtzensis* is circulated and *Rattus* spp. and *I. granulatus* play the roles of the natural reservoir and vector, respectively, in Sarawak. However, there is a limitation in our study; as all of the positive *Ixodes* ticks were engorged, we could not rule out whether borrelial DNA detected from the tick samples was from the blood meal host or not. Thus, further investigations of *B. yangtzensis* in unfed *I. granulatus* are required to confirm the vector species of this bacterium in Sarawak. In addition, *I. granulatus* has been documented from migratory birds in Taiwan by Kuo et al. [41]. Migratory birds are known to play an important role in the dispersal of Bbsl, with previous reports involving *Ixodes* ticks from Japan, South Korea, and Russia [42,43,44]. Moreover, studies in Canada by Scott et al. showed that migratory birds disperse Bbsl-infected ticks over a long distance and across geographical barriers [45,46]. Thus, investigation of ticks collected from migratory birds in Malaysia might help expand our knowledge of Bbsl, including *B. yangtzensis*.

Multilocus sequence analysis was first introduced by Margos et al. [47] for depicting the evolutionary processes of *B. burgdorferi.* By targeting multi loci, eight housekeeping genes were developed for the Bbsl complex [47] and have been subsequently used in other studies to characterize the complexity of the Bbsl genospecies [33,35]. In recent years, this method has also proven to be useful in comparing the intraspecific diversity, elucidating population genetic structure, and other ecological aspects that may contribute to the transmission dynamics of the Bbsl genospecies [48,49]. Further, MLSA has been used to confirm *B. yangtzensis* from the isolates of ticks and rodents from China and Japan [33]. Based on MLSA, the phylogeny inference revealed that the isolates formed two sister clusters, with each cluster consisting of isolates from both China and Japan. Concordantly, in our study, the concatenated sequences of *B. yangtzensis* from *I. granulatus* were also located in two sister clades.

On the human pathogenic aspect, *B. valaisiana* had been regarded as the causative agent of LD in humans but was recently proven otherwise [50]. Two human LD cases caused by *B. valaisiana*-related genospecies were reported from Japan and China [51,52], but Margos et al. [33,50] later on ratified them be *B. yangtzensis* and suggested *B. valaisiana* as a non-human pathogenic. Although *B. yangtzensis* may potentially be a human pathogenic, there is currently no study providing further evidence. Even though the significance of *B. yangtzensis* in humans and animals is not yet fully understood, the findings of *B. yangtzensis* in our study imply the likelihood that the bacterium circulates within the *Ixodes* ticks and rodents in primary forests and OP plantation in Sarawak. *Borrelia* spp. closely related to *B. yangtzensis* were also detected from *I. granulatus* in Peninsular Malaysia [26]. Of note, *I. granulatus* is a rare parasite of humans with few related reports. Yun et al. [53] identified only one female of *I. granulatus* from 261 ticks they collected from humans in South Korea. A checklist of ticks from Thailand, dated back to 1983, documented that humans could be hosts for this tick species [54]. However, it was not clear in these studies whether the *I. granulatus* collected were biting humans. Thus, more investigations are required to evaluate the pathogenicity to humans and understand the transmission cycle of *B. yangtzensis* in Malaysia.

*Borrelia miyamotoi*, the causative agent of RF, was first isolated from *I. persulcatus* ticks in Japan. To date, several *Ixodes* tick species are considered as a vector of *B. miyamotoi*. For instance, *I. scapularis* and *I. pacificus* are the vectors reported in the United States and Canada, *I. ricinus* in Europe, and *I. persulcatus* in Europe and Asia [16,23,55,56,57]. So far, the reservoir hosts, based on the geographical distribution, for *B. miyamotoi* are still not well understood, but rodents and birds have been considered as the reservoir hosts in some regions [58,59]. In this study, we detected *B. miyamotoi* from *S. muelleri* in GGNP; this is the first report of *B. miyamotoi* in Malaysia. However, none of the *I. granulatus* examined in this study were positive for *B. miyamotoi*. Furthermore, the infection rate of *B. miyamotoi* was lower than that of *B. yangtzensis* in this study. Generally, the infection rate of *B. miyamotoi* in rodents and ticks appears to be lower than that of Bbsl species as per previous studies in Japan and Russia [56,58]. Further, another study on the prevalence of *B. miyamotoi* infection in *I. scapularis* conducted in Canada was low (<1%) [57]. Moreover, the reported prevalence of *B. miyamotoi* in questing *Ixodes* ticks ranged from 1.3% in *I. Ricinus* to 3.6% in *I. persulcatus* [60]. For future studies, the sample size of the rodents and ticks should be increased to find the vector tick species and to describe the diversity and distribution of *B. miyamotoi* in Sarawak. In addition, *B. miyamotoi* has been recently reported from *H. concinna* in Northeastern China [23]. In Europe, migratory birds have been reported as the reservoir host of *B. miyamotoi* or play a role in the dispersal of tick vectors [59]. Thus, the investigations of other tick species and birds may provide more in-depth insights of *B. miyamotoi* in Sarawak.

The sampling in this study was conducted only once for the primary forests (GGNP and KNP) and the OP plantation in different seasons, which yielded a small sample size, especially in the sampling during the wet season. A small sample size and lack of sampling repetition may have contributed to the low number of positive samples in this study; as *B. miyamotoi* was only positive in one rodent, and *B. yangtzensis* was not detected in the rodents from GGNP and KNP. In addition, only rodent spleens were used in this study for Bbsl and RFB screening. Future work to estimate the prevalence should include ear biopsies and other internal organs, as Bbsl and *B. miyamotoi* may not have the same strategies for maintenance and dissemination in the same reservoir host [61]; therefore, different tissue may yield different detection rate [62]. Despite the incomparable rodent numbers, the number of *Ixodes* ticks from the primary forests and OP plantation were fairly similar (14 and 18, respectively). In the OP plantation, 13 ticks were positive for *B. yangtzensis*; in contrast, in the primary forests, only one tick from KNP was positive. Land conversion with a human-dominated ecosystem could have a potent effect on reservoirs and the zoonotic risk, because of the alterations of host diversity and composition [63,64]. The difference observed in this study might be reflected by the variation between the primary forest and OP plantation. A follow-up study to evaluate this hypothesis should encompass a larger sampling size with repetition.

In conclusion, we examined *Borrelia* spp. in rodents and ticks from primary forests and an OP plantation. We reported the presence of *B. miyamotoi* for the first time in Malaysia. We also reported the first detection of *B. yangtzensis*, which was characterized using MLSA, in both rodents and *I*. *granulatus* in Sarawak. These findings of *Borrelia* spp. in Sarawak provide evidence of a new geographical record. Our study warrants the need for further investigations as it is important to determine how the *Borrelia* spp. may impact public health in Malaysia.

## 4. Materials and Methods

### 4.1. Ethics Approvals

The collecting of rodents and ticks was approved by the Sarawak Forests Department, Malaysia (Permit No. (91) JHS/NCCD/600-7/2/107 and Park Permit No. WL47/2018; Permit No. (11) JHS/NCCD/600-7/2/107(Jld2) and Park Permit No.WL5/2019). The sampling methods were approved by the Animal Care and Use Committee of Hokkaido University, Japan (Approval No. 18-0081). The samples were exported with the permission of the Sarawak Forests Department (No.18650).

### 4.2. Survey Sites and Sample Collection

We selected the protected primary forests, GGNP (1.69° N, 109.85° E) and KNP (1.61° N, 110.20° E), and an OP plantation (3.36° N, 113.69° E) in Sarawak as the study sites. The rodents and ticks attached to the rodent hosts were collected from GGNP and KNP during the wet season in November 2018 and from the OP plantation during the dry season in March 2019 (Figure 1; Table 1). The sampling period for each site ranged from 5–10 days. The rodents were captured using collapsible cage traps; their tentative species, sex, breeding status, and body measurements were recorded. The captured rodents were individually anesthetized using isoflurane. Next, the ticks attached to each rodent were collected and kept separately in 70% ethanol. In addition, the selected rodents were euthanized following the method described by Taylor et al. [58] for internal organs collection. Finally, the harvested organs were kept in 70% ethanol and subsequently stored at −20 °C after being transferred back to the facility; the spleen samples of the rodents were used in this study.

### 4.3. DNA Preparation and Species Identification of Rodents and Ticks

DNA was extracted from the rodent spleens at the Faculty of Resource Science and Technology, Universiti Malaysia Sarawak, using a Wizard^®^ Genomic DNA Purification Kit (Promega, Madison, WI, USA), following the manufacturer’s instructions. The DNA samples and ticks were sent to Hokkaido University, Japan, where the subsequent screenings and analyses of the samples were conducted.

For the molecular identification of the rodent species, we amplified a fragment of the cytochrome c oxidase subunit 1 (*CO1*) by polymerase chain reaction (PCR) using the primer pairs BatL5310 and R6036R (Table 3) [65]. The PCRs were conducted in a 20 µL reaction mixture using the *Ex Taq* Hot Start version (Takara Bio, Shiga, Japan) with the following conditions: 30 cycles of denaturation at 94 °C for 30 s, annealing at 48 °C for 30 s, and extension at 72 °C for 60 s.

The tick genera or species were morphologically identified based on the taxonomic keys [66,67], and a fragment of their mitochondrial 16S ribosomal DNA (16S rDNA; Table 3) was confirmed by sequencing [68]. One leg of the ticks was removed, and DNA was extracted from it by using the hot alkaline extraction method previously described by Mtambo et al. [69], with some modifications. Briefly, we added 10 µL of 100 nM of sodium hydroxide and incubated it at 95 °C for 10 min, followed by adding 2 µL of tris-hydrochloride buffer (pH 7.0). The PCR was conducted using Tks Gflex DNA Polymerase (Takara Bio, Shiga, Japan), with the following conditions: initial denaturation at 94 °C for 1 min, followed by 40 cycles of 98 °C for 10 s, 55 °C for 15 s, and 68 °C for 24 s, and a final extension at 68 °C for 5 min.

The amplification products from rodents and ticks were electrophoresed on a 1.2% agarose gel with Midori Green Direct DNA stain (Nippon Genetics, Tokyo, Japan) and visualized with a BLooK LED transilluminator (GeneDireX, Las Vegas, NV, USA). The Sanger sequencing was performed using the BigDye Terminator version 3.1 Cycle Sequencing Kit (Applied Biosystems, Foster City, CA, USA). The sequencing products were analyzed on an ABI Prism 3130 x genetic analyzer (Applied Biosystems, Foster City, CA, USA), according to the manufacturer’s instructions. The sequences were compared with public databases using the Nucleotide Basic Local Alignment Search Tool (BLASTn) (https://blast.ncbi.nlm.nih.gov/Blast.cgi). In addition, the Barcode of Life Data System (BOLD; http://www.barcodinglife.org) was also used for rodent identification [70].

After identifying the tick species, the remaining body of the *Ixodes* ticks was washed with sterile phosphate-buffered saline and individually crushed with Micro Smash MS-100R (TOMY, Tokyo, Japan) for 30 s at 2500 rpm. The DNA was extracted using the Wizard^®^ Genomic DNA Purification Kit (Promega, Madison, WI, USA), as described above.

### 4.4. Screening of the Borrelia spp.

DNA from the rodent spleens and *Ixodes* ticks were subjected to the screening of Bbsl and RFB using a nested PCR targeting the *flaB*, which is a 345 bp amplicon (Table 3) [71]. The PCR conditions were as follows: 25 and 30 cycles of denaturation at 94 °C for 30 s, 55 °C and 50 °C of annealing for 30 sec, and extension at 72 °C for 1 min in the first and second PCR, respectively. The positive samples of the *flaB*-PCR were further characterized by additional PCRs, targeting 16S rDNA, which is approximately a 1370 bp amplicon. For the *Ixodes* ticks, a single PCR with BF1 and BR1 primers was performed [73] (Table 3). While, for the rodent samples, universal primers targeting bacterial 16S rDNA were added for the first PCR [72], BF1 and BR1 primers were added for the second PCR. The PCR conditions for the single and nested PCRs were identical, i.e., 35 cycles of denaturation at 94 °C for 30 s, annealing at 55 °C for 30 s, followed by extension at 72 °C for 90 s. The PCR was conducted using the *Ex Taq* Hot Start version (Takara Bio, Shiga, Japan) with a reaction mixture of 20 µL. DNA from “*Candidatus* Borrelia fainii” strain Qtaro [74] and molecular-grade water were used for the positive and negative controls, respectively. Finally, the electrophoresis, PCR product purification, and Sanger sequencing were performed as mentioned in the subsection “DNA preparation and species identification of rodents and ticks”.

### 4.5. Multilocus Sequence Analysis of the Borrelia spp.

Four rodent and six tick samples were randomly selected from the *flaB* and 16S rDNA PCR positive samples and were used for MLSA based on the sequences of eight housekeeping genes (*clpA*, *clpX*, *nifS*, *pepX*, *pyrG*, *recG*, *rplB*, and *uvrA*). In order to obtain the sequences from these genes, we employed previously described methods with slight modification [47]. Briefly, nested PCRs using *Ex Taq* Hot Start version (Takara Bio, Shiga, Japan) were performed without the touchdown step, initially. Then, for the samples that failed to amplify, we repeated the PCR with the touchdown step. Finally, the PCR products were observed with gel electrophoresis and purified using a FastGene Gel/PCR Extraction Kit (Nippon Genetics, Tokyo, Japan), followed by Sanger sequencing.

### 4.6. Sequencing and Phylogenetic Analyses

The sequences were assembled and trimmed using the ATGC software version 6.0.4 (GENETYX, Tokyo, Japan) and compared with the sequences available in public databases using BLASTn. The phylogenetic trees were constructed using MEGA version X [75] with Neighbor-Joining or maximum likelihood models and the Kimura 2-parameter model with pairwise deletion and 1000 bootstrap replications. The phylogenetic relationships of the *Borrelia* spp. were also analyzed using the concatenated sequences of the eight genes and those of closely related *Borrelia* spp. downloaded from the PubMLST database (https://pubmlst.org/). The sequences obtained in this study are available in the GenBank database with following accession numbers: *flaB* (LC572294–LC572312), 16S rDNA (LC572071–LC572081), *clpA* (LC572082–LC572087), *clpX* (LC572088–LC572095), *nifS* (LC572096–LC572101), *pepX* (LC572102–LC572111), *pyrG* (LC572112–LC572119), *recG* (LC572120–LC572128), *rplB* (LC572129–LC572136), and *uvrA* (LC572137–LC572145).

## Figures and Tables

**Table 1:** The number of rodent species and *Ixodes granulatus* used for the screening of *Borrelia* spp.

	Sampling Period			
	November 2018		March 2019	
Rodent Species	GGNP	KNP	OP Plantation	Total
*Leopodamys sabanus*	0/2	0/2	N/A	0/4
*Maxomys rajah*	N/A	0/2	N/A	0/2
*Maxomys whiteheadi*	0/2	N/A	N/A	0/2
*Rattus* spp.	N/A	0/3	4/42	4/45
*Sundamys muelleri*	1/2	N/A	0/1	1/3
**Total**	1/6	0/7	4/43	5/56
***Ixodes granulatus***	**GGNP**	**KNP**	**OP Plantation**	**Total**
Female	0/3	1/9	10/10	11/22
Nymph	N/A	0/2	1/3	1/5
Larva	N/A	N/A	2/5	2/5
**Total**	0/3	1/11	13/18	14/32

No. of positive/No. of tested. Gunung Gading National Park (GGNP), Kubah National Park (KNP), oil palm (OP).

**Table 2:** The sequence analysis results for the borrelial flagellin gene (*flab*) and 16S ribosomal DNA (rDNA) of *Ixodes* ticks.

Gene	Sample ID	BLASTn	Identity	Accession No.
***flaB***	IG-204	*Borrelia yangtzensis* strain QTMP2 (China)	100% (300/300 bp)	EU135602
	IG-206	*Borrelia yangtzensis* strain QTMP2 (China)	100% (300/300 bp)	EU135602
	IG-208	*Borrelia yangtzensis* strain QTMP2 (China)	100% (300/300 bp)	EU135602
	IG-213	*Borrelia yangtzensis* strain QTMP2 (China)	100% (300/300 bp)	EU135602
	IG-217	*Borrelia yangtzensis* strain QTMP2 (China)	100% (300/300 bp)	EU135602
	IG-218	*Borrelia yangtzensis* strain QTMP2 (China)	100% (300/300 bp)	EU135602
	IG-215	*Borrelia valaisiana*-related genospecies (Japan)	99.3% (298/300 bp)	AB091710
	IG-219	*Borrelia valaisiana*-related genospecies (Japan)	100% (300/300 bp)	AB091710
	IG-214	*Borrelia* sp. TKM-30 from *Ixodes granulatus* (Taiwan)	99.3% (298/300 bp)	HM853004
	IG-216	*Borrelia* sp. TKM-30 from *Ixodes granulatus* (Taiwan)	99.3% (298/300 bp)	HM853004
	IG-220	Uncultured *Borrelia* sp. clone Borr65 from *Ixodes granulatus* (China)	100% (300/300 bp)	MG717514
	IG-221	Uncultured *Borrelia* sp. clone BorrIg from *Ixodes granulatus* (China)	99.0% (297/300 bp)	MG717513
	IG-222	Uncultured *Borrelia* sp. from *Ixodes granulatus* (Malaysia)	99.7% (299/300 bp)	LT969779
	IG-228	Uncultured *Borrelia* sp. from *Ixodes granulatus* (Malaysia)	100% (300/300 bp)	LT969779
**16S rDNA**	IG-213	*Borrelia yangtzensis* strain QX-S13 (China)	99.5% (1347/1354 bp)	EU135598
	IG-216	*Borrelia* sp. 9MT (South Korea)	99.8% (1351/1354 bp)	L39080
	IG-217	*Borrelia valaisiana*-related genospecies from rodent *Apodemus agrarius* (China)	99.6% (1348/1354 bp)	AB022140
	IG-220	*Borrelia valaisiana*-related genospecies from rodent *Apodemus agrarius* (China)	99.6% (1346/1352 bp)	AB022140
	IG-218	*Borrelia yangtzensis* strain QSYSP3 (China)	99.8% (1351/1354 bp)	EU135595
	IG-219	*Borrelia valaisiana*-related genospecies from rodent *Apodemus agrarius* (China)	99.7% (1350/1354 bp)	AB022141
	IG-221	*Borrelia valaisiana*-related genospecies from rodent *Apodemus agrarius* (China)	99.6% (1349/1354 bp)	AB022141

**Table 3:** The primers used in this study.

Primer Name	Sequence (5′ to 3′)	Target Gene (PCR Type)	Annealing Temperature (°C)	Amplicon Size (bp)	Reference
mt-rrs1	CTGCTCAATGATTTTTTAAATTGCTGTGG	Mitochondrial 16S rDNA of tick (Single PCR)	55	~400	[68]
mt-rrs2	CCGGTCTGAACTCAGATCAAGTA				
BatL5310	CCTACTCRGCCATTTTACCTATG	*CO1* of rodents (Single PCR)	48	750	[70]
R6036R	ACTTCTGGGTGTCCAAAGAATCA				
BflaPAD	GATCARGCWCAAYATAACCAWATGCA	*flaB* of *Borrelia* (1st PCR)	55	800	[71]
BflaPDU	AGATTCAAGTCTGTTTTGGAAAGC				
BflaPBU	GCTGAAGAGCTTGGAATGCAACC	*flaB* of *Borrelia* (2nd PCR)	50	345	[71]
BflaPCR	TGATCAGTTATCATTCTAATAGCA				
fD1	AGAGTTTGATCCTGGCTCAG	Universal primer for 16S rDNA of bacteria (1st PCR for rodent samples)	55	1400	[72]
rp2	ACGGCTACCTTGTTACGACTT				
BF1	GCTGGCAGTGCGTCTTAAGC	16S rDNA of *Borrelia* (Single PCR for tick and 2nd PCR for rodent samples)	55	1371	[73]
BR1	GCTTCGGGTATCCTCAACTC				
* BF3_seq	AGATACCCTGGTAGTCTACGCT	16S rDNA of *Borrelia*	N/A	N/A	This study
* BR3_seq	GCTGCTGGCACGTAATTAGC				

* Primers were used in sequencing.

## Supplementary Materials

The following are available online at https://www.mdpi.com/2076-0817/9/10/846/s1, Figure S1: Phylogenetic inference of *Borrelia burgdorferi* sensu lato included rodent samples, based on individual housekeeping genes. (A) *clpX*, (B) *pepX*, (C) *pyrG*, (D) *recG*, (E) *rplB*, (F) *uvrA*.
