# Supplementary figures and images for: Detection of Borrelia burgdorferi Sensu Lato and Relapsing Fever Borrelia in Feeding Ixodes Ticks and Rodents in Sarawak, Malaysia: New Geographical Records of Borrelia yangtzensis and Borrelia miyamotoi
Source: Pathogens. 2020 Oct 15;9(10):846. doi: 10.3390/pathogens9100846 (PMC7650685; doi:10.3390/pathogens9100846)

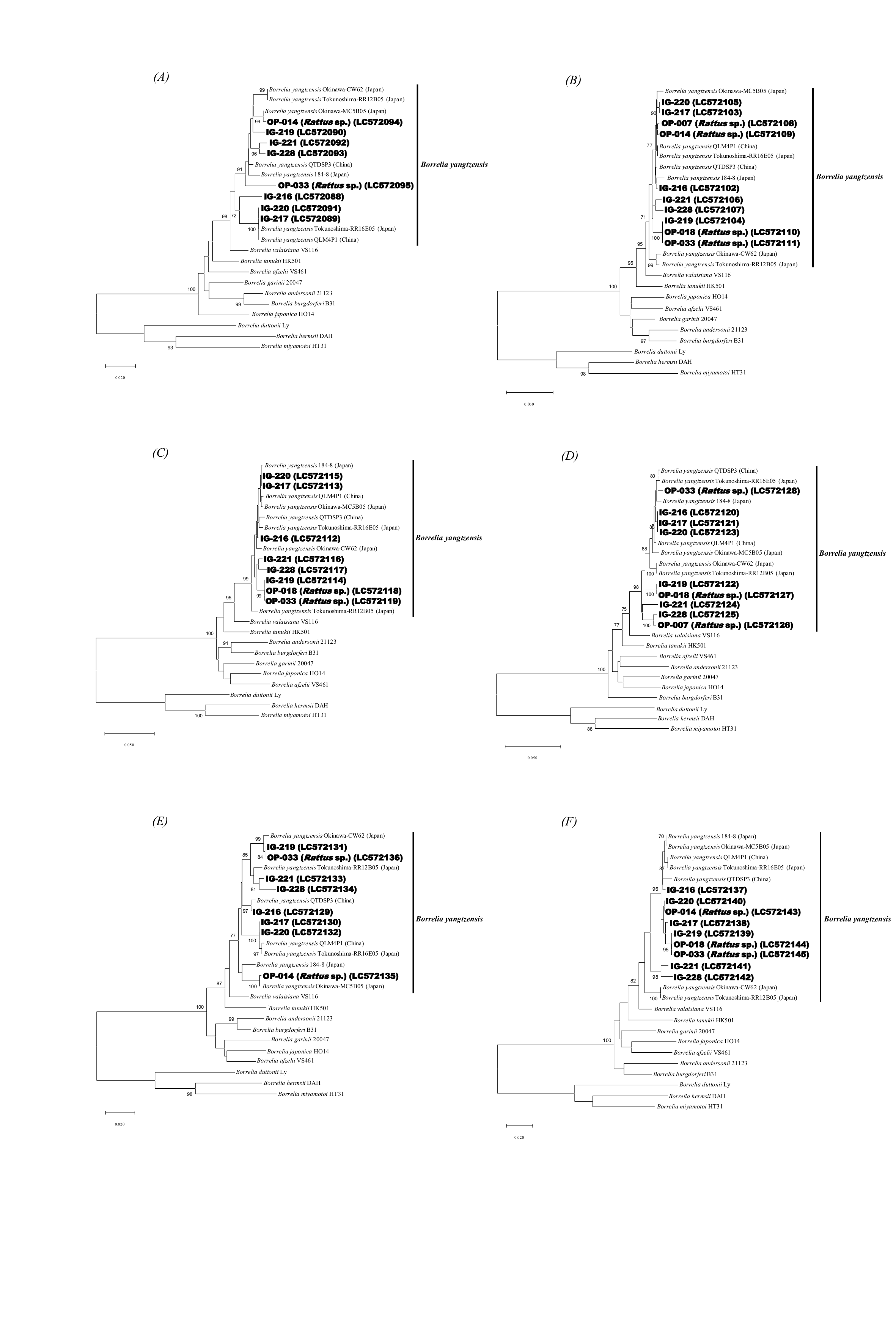

Supplement: Supplementary file 1 [file pathogens-09-00846-s001.zip › FigureS1.tif]
